# A novel tablet-based software for the acquisition and analysis of gaze and eye movement parameters: a preliminary validation study in Parkinson’s disease

**DOI:** 10.3389/fneur.2023.1204733

**Published:** 2023-06-15

**Authors:** Étienne de Villers-Sidani, Patrice Voss, Daniel Guitton, J. Miguel Cisneros-Franco, Nils A. Koch, Simon Ducharme

**Affiliations:** ^1^Innodem Neurosciences, Montreal, QC, Canada; ^2^Montreal Neurological Institute, McGill University, Montreal, QC, Canada; ^3^Integrated Program in Neuroscience, McGill University, Montreal, QC, Canada; ^4^Douglas Mental Health University Institute, Montreal, QC, Canada

**Keywords:** eye-tracking, biomarker, Parkinson’s disease, UPDRS (unified Parkinson’s disease rating scale), gaze mapping, neurodegenerative disorders

## Abstract

The idea that eye movements can reflect certain aspects of brain function and inform on the presence of neurodegeneration is not a new one. Indeed, a growing body of research has shown that several neurodegenerative disorders, such as Alzheimer’s and Parkinson’s Disease, present characteristic eye movement anomalies and that specific gaze and eye movement parameters correlate with disease severity. The use of detailed eye movement recordings in research and clinical settings, however, has been limited due to the expensive nature and limited scalability of the required equipment. Here we test a novel technology that can track and measure eye movement parameters using the embedded camera of a mobile tablet. We show that using this technology can replicate several well-known findings regarding oculomotor anomalies in Parkinson’s disease (PD), and furthermore show that several parameters significantly correlate with disease severity as assessed with the MDS-UPDRS motor subscale. A logistic regression classifier was able to accurately distinguish PD patients from healthy controls on the basis of six eye movement parameters with a sensitivity of 0.93 and specificity of 0.86. This tablet-based tool has the potential to accelerate eye movement research via affordable and scalable eye-tracking and aid with the identification of disease status and monitoring of disease progression in clinical settings.

## Introduction

While we have long known that the eyes are our windows to the world, a growing body of research suggests a bi-directional relationship whereby the eyes –and particularly how they move– can also serve as a window into the brain. Although eye movements have previously been linked to certain cognitive processes like attention and decision-making, recent work has unequivocally shown that eye movements can reflect certain aspects of brain function and inform on the presence of neurodegeneration and cognitive impairment (1–5). The link between eye movements and brain health should not be too surprising given that eye movements are controlled by a diverse network of cortical and subcortical structures (4, 6) that are susceptible to a variety of degenerative processes (1, 7, 8). Moreover, the analysis of gaze patterns and visual tasks that measure cognitive inhibition can provide insights into the integrity of various cognitive processes (2, 9, 10).

For instance, two consistent impairments have emerged from Alzheimer’s disease (AD) oculomotor research: a high frequency of saccadic intrusions during attempted fixation and visual capture by the target in the anti-saccade paradigm (11, 12). Furthermore, microsaccades, tiny horizontal rapid eye movements that interrupt periods of fixation tend to be uniquely obliquely oriented (13) and occur at an elevated rate in Alzheimer’s (14). Parkinson’s Disease (PD) is generally associated with hypometric and multi-step saccades in all types of oculomotor tasks (15, 16), in addition to a high rate of saccadic intrusions during smooth pursuit (17). Multiple sclerosis (MS) is particularly associated with internuclear ophthalmoparesis (INO)—a slowing of the adducting eye during horizontal saccades—and saccadic intrusions during fixation (8). Furthermore, smooth pursuit metrics (low pursuit gain and increased saccadic amplitudes) have been proposed as a marker of early MS (18).

Not only do these oculomotor anomalies characterize these neurodegenerative disorders, but a growing body of research shows that they can serve as markers of disease severity and cognitive impairment. In AD, oculomotor signatures of disease severity have been identified via correlations between specific eye movement characteristics and the Mini-Mental State Examination (MMSE) (19–22). Similarly, in PD, several oculomotor metrics have been shown to correlate with the Unified Parkinson’s Disease Rating Scale (UPDRS) or some of its subscales (23–26), or with measures of general cognition such as the MMSE (27, 28) or the MoCA (29, 30). In MS similar relationships have been observed between such metrics and the Expanded Disability Status Scale (EDSS) or the Symbol Digit Modalities Test (SDMT) (31–35).

Although a clinical oculomotor examination is usually sufficient to aid clinicians in the differential diagnosis of advanced neurological disorders, these exams do not typically capture subtle changes such as those highlighted in the aforementioned studies. Indeed, many have proposed that laboratory eye movement recordings can be extremely useful for objective and precise identification of disease status and monitoring of disease progression (1) and assist with differential diagnoses (36–38) though there is hope that the precise quantification of eye movements could also eventually lead to early diagnoses in individuals with less pronounced oculomotor symptoms. Unfortunately, the use of detailed eye movement recordings in clinical settings has been limited due to the expensive nature and limited scalability of the required equipment, such as infrared eye-tracking cameras. Although several mobile tablet-based (or smartphone-based) gaze-tracking systems have been developed to provide more accessible and affordable solutions (39–42), to our knowledge, they have not been used to capture precise oculomotor parameters on a millisecond timescale such as those evoked during saccade and anti-saccade tasks. They have instead been primarily used to analyze gross eye movements, such as those required to study gaze search patterns or to determine the on-screen location of an individual’s fixation point.

Eye-Tracking Neurological Assessment (ETNA™) is a recently developed technology that can reliably and accurately track eye movements without the need for infrared cameras, using the iPad Pro embedded camera. This technology allows for the precise quantification of several eye movement parameters currently only available with specialized and costly research-grade infrared eye tracking devices, such as the latency, velocity, and amplitude of saccades, and the presence of saccadic intrusions during fixation. In this paper, we show using the ETNA™ with a standard tablet mobile camera that we can measure and replicate eye movement anomalies and replicate findings from the literature on PD and eye movement, further demonstrating how eye movement parameters can reflect disease status and severity.

## Methods

### Study design and subject population

This cross-sectional study included 121 participants and was approved by both the Veritas and the Montreal University Health Center (MUHC) research ethics boards. Fifty-nine (59) PD participants (age 63.76 ± 8.23, range 45–79, 32.2% females) took part in this study. All were recruited by the Quebec Parkinson Network. Inclusion criteria were confirmed diagnosis of PD and sufficient corrected visual acuity to allow for the accurate reading of the on-screen visual task instructions. Exclusion criteria were the presence of comorbid neurological or psychiatric conditions to avoid eye movement anomaly confounds. To assess clinical status, all PD patients underwent the motor subscale (part III) of the MDS-UPDRS (43, 44), which was developed to evaluate various aspects of Parkinson’s Disease. Note that because the MDS-UPDRS was performed in a research setting with time constraints and not as part of the standard of care, not all patients underwent the full MDS-UPDRS evaluation. As a result, only part III scores were used in analyses presented herein.

Sixty-two (62) healthy control (HC) participants (age 56.64 ± 8.56, range 45–77, 46.7% females) took part in this study. All were recruited from the Montreal community. The inclusion criterion was sufficient visual acuity to perform the tablet-based visual tasks. Exclusion criteria were evidence or history of other significant neurological or psychiatric disorders. Summary patient demographics are shown in Table 1.

**Table 1:** Group demographics.

	PD patients	Healthy controls
*n*	59	62
Age mean ± sd (median, range)	63.76 ± 8.23 (64, 45–79)	56.64 ± 8.56 (55; 45–77)
UPDRS part III	27.56 ± 13.8 (7–65)	

### Gaze-tracking experimental setup

**Figure 1:**
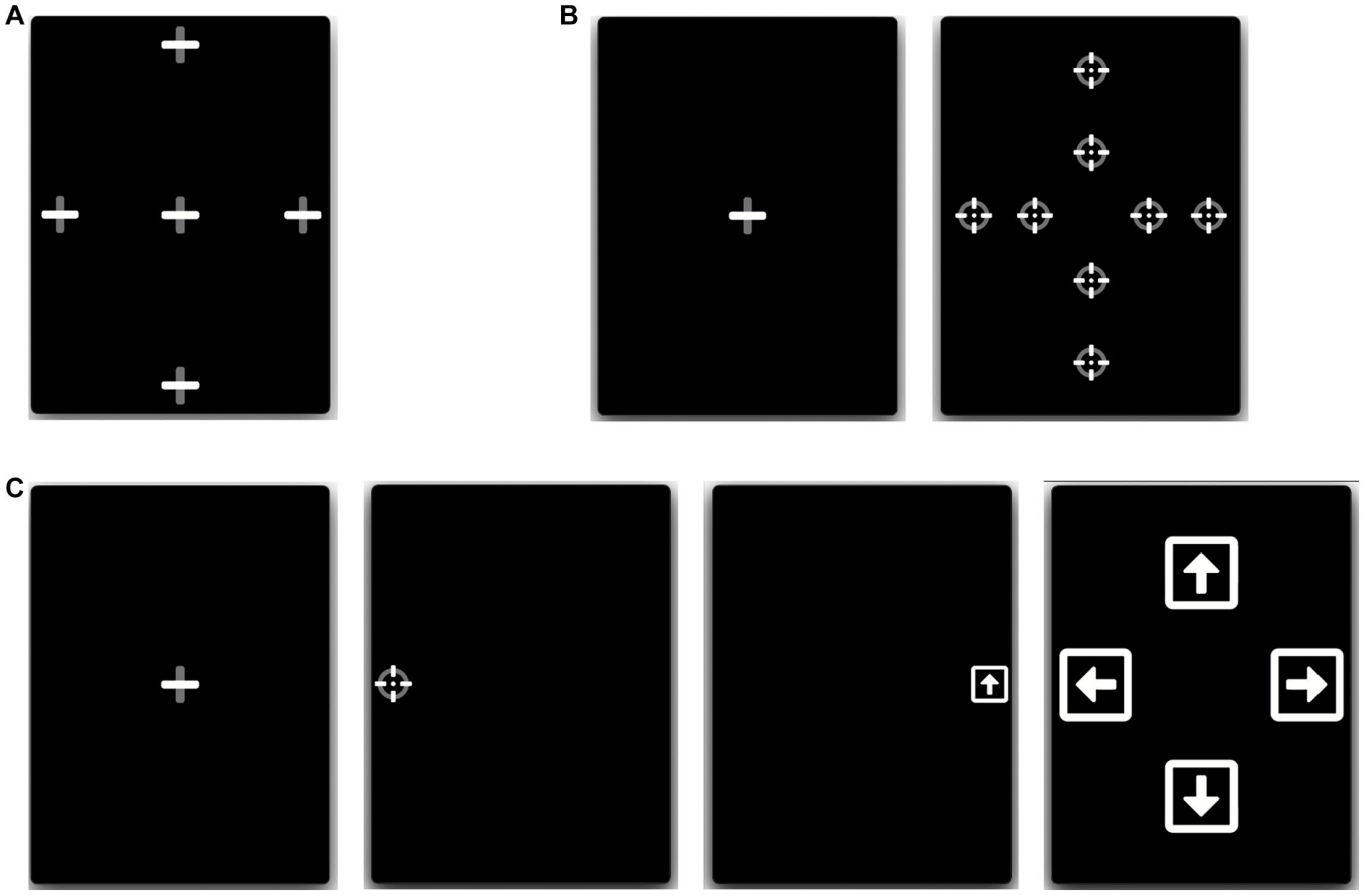
Eye-tracking tasks. **(A)** Fixation: participants fixated a stationary target for 7 s, at one of 5 locations. **(B)** Pro-saccades: participants initially fixated a central fixation point, which disappeared after 1.0–3.5 s, after which a different target appeared at one of 8 eccentric locations for 1.5 s. **(C)** Anti-saccades: participants initially fixated a central fixation point, which disappeared after 1.0–3.5 s, after which a round target appeared at 10° to the left or right from the center. Participants were instructed to move their gaze in the opposite direction to the round target, where after 1,200 ms they were shown a square with an arrow inside that pointed in one of 4 random directions (left, right, up, or down; shown during 400 ms). The users then had to direct their gaze towards the arrow orientation corresponding to the arrow they saw in the preceding step.

All tests were performed using a 12.9-inch iPad Pro tablet with the ETNA™ software installed, which enables simultaneous video recordings of the eyes at 60 frames per second using the embedded front-facing camera and the presentation of visual stimuli on the screen. All participants performed three oculomotor tasks (fixation task, pro-saccade task, and anti-saccade task; see Figure 1), which was preceded by a calibration step, where participants were instructed to follow a slowly moving target across the screen. All tablet-based oculomotor tasks were completed in under 6 min.

All tasks were performed with the tablets placed vertically, camera side up, and secured at eye level using a tablet pole mount. Participants were positioned approximately 45 cm from the tablet screen and were allowed to use their best-spectacle correction (proportion of participants wearing glasses: PD = 39%, HC = 29%, *X*^2^(1) = 1.33, *p* = 0.24). Safeguards within the gaze-tracking software ensured the participant’s head was properly positioned and visible, at an acceptable angle and distance from the screen.

**Fixation task:** Participants had to fixate a stationary target for 7 s, at five different locations (one central and 4 eccentric locations). The eccentric positions were located 10 degrees of visual angle left and right from the center and 14 degrees of visual angle up and down from the center (Figure 1A).

**Pro-saccade task:** Participants had to initially fixate a central fixation point, which disappeared after a random period of 1.0–3.5 s, after which a different target reappeared at an eccentric location for 1.5 s either to the left or right, above, or below the central fixation point. Participants were instructed to move their gaze as quickly as possible to the new target location. Both short (5^o^ horizontal, 6^o^ vertical) and large (10^o^ horizontal, 12^o^ vertical) eccentric target distances were used, and each target location was sampled 3 times, for a total of 24 trials (Figure 1B).

**Anti-saccade task:** Participants had to initially fixate a central fixation target, which disappeared after a random period of 1.0–3.5 s, after which a different target reappeared at an eccentric location (10^o^) to the left or right from the center. Participants were instructed to move their gaze as quickly as possible in the opposite direction to the new target location. After being displayed for only 100 ms, the target disappeared and the screen was left blank for a predetermined duration of time. Following the blank screen, a symbol appeared in the opposite location of where the initial stimulus appeared (i.e., where the participant should be looking). This symbol consisted of a white square with an arrow inside oriented in one of 4 random directions: either left, right, up, or down. The blank screen period lasted 1,200 ms and the arrow symbol duration of 400 ms. After each trial, a screen was displayed for 5 s prompting the user to answer which symbol they saw by directing their gaze towards the arrow orientation corresponding to what they believe is the correct answer (Figure 1C). This task was inspired by an anti-saccade task used in a previous study (45), whereby participants could only identify the second symbol had they performed the anti-saccade task correctly (i.e., looked in the opposite direction of the initial target).

### Parameter extraction and analysis

Offline analysis was performed using ETNA™‘s proprietary analysis pipeline to automatically extract the eye movement parameters reported for each task. Before parameter extraction, all gaze signals were denoised and non-saccadic artifacts (e.g., blinks) were removed by the software’s analysis pipeline.

The following parameters were extracted from the *fixation task* gaze recordings (parameters were averaged across the five fixation trials): (1) 68% bivariate contour ellipse area (BCEA) of fixation – a measure of fixation stability which encompasses an ellipse that covers the 68% of fixation points that are closest to target, (2) 95% BCEA, (3) Horizontal gaze SD – standard deviation of the horizontal gaze position, (4) Vertical gaze SD – standard deviation of the vertical gaze position, and (5) the rate of saccadic intrusions (at least 0.5 deg. in amplitude) during fixation.

The following parameters were extracted from the *pro-saccade task* gaze recordings (averaged across all short-eccentricity targets and all large-eccentricity targets): (1) average saccade latency, (2) average total time to reach the target, (3) average mean saccade velocity, (4) average peak saccade velocity, (5) average saccade amplitude gain (amplitude of the saccade relative to the eccentricity of the target; a measure of saccade accuracy), (6) average saccade amplitude error (average distance separating the saccade from the target; a measure of saccade precision), and (7) the average number of saccades required to reach the target.

The following parameters were extracted from the *anti-saccade task* gaze recordings: (1) direction error rate, (2) direction corrected rate (proportion of trials where participants directed their gaze in the correct direction following an initial saccade in the wrong direction), (3) target (arrow) recognition rate, (4) correct direction latency, and (5) incorrect direction latency.

Group comparisons were performed using multivariate analysis of covariance to simultaneously test statistical differences for multiple response variables (eye-tracking parameters) by one grouping variable (PD or HC). As age and sex were significantly different between study groups (t(1) = 4.66, *p* < 0.001), these grouping variables were used as covariates for between-group comparisons. *F*-statistics with degrees of freedom and *p*-values are reported. For correlation analyses with MDS-UPDRS-part III (motor) scores, data normality was assessed with the Shapiro–Wilk test to determine the appropriate correlation coefficient for each eye-movement parameter (i.e., Pearson’s R or Spearman’s *ρ*). Data analyses and visualization were conducted using R 4.2.1 in RStudio (build 554), packages *dplyr*, *tidyverse*, *ggplot2*, *ggpubr*, and *rstatix*. Although the main purpose of the present paper is to replicate well-known findings in the literature using a novel device, and not to make novel scientific claims, we opted for transparency to present corrected *p*-values for correlations and post-hoc between-group comparisons to adjust for the false discovery rate using the Benjamini-Hochberg procedure evaluated at an alpha level of 0.05 (46).

Finally, a logistic regression with ridge regularization and random subsampling (1,000 samples) is used to assess the strength of six eye-tracking parameters [the fixation saccadic intrusion rate (1), for short amplitude pro-saccades: the first gain (2), mean velocity (3), mean latency (4) and the average number of saccades (5), and the first gain error for large amplitude pro-saccades (6)] as predictors of PD diagnosis (PD vs. HC). Receiver Operating Characteristics (ROC) analysis and a confusion matrix were used to assess the performance of the classifier. Training, classification and visualization of the logistic regression classifier was conducted using scikit-learn 1.2.2 and matplotlib 3.7.1 in Python 3.9.6.

## Results

### Correlations with MDS-UPDRS – part III

**Figure 2:**
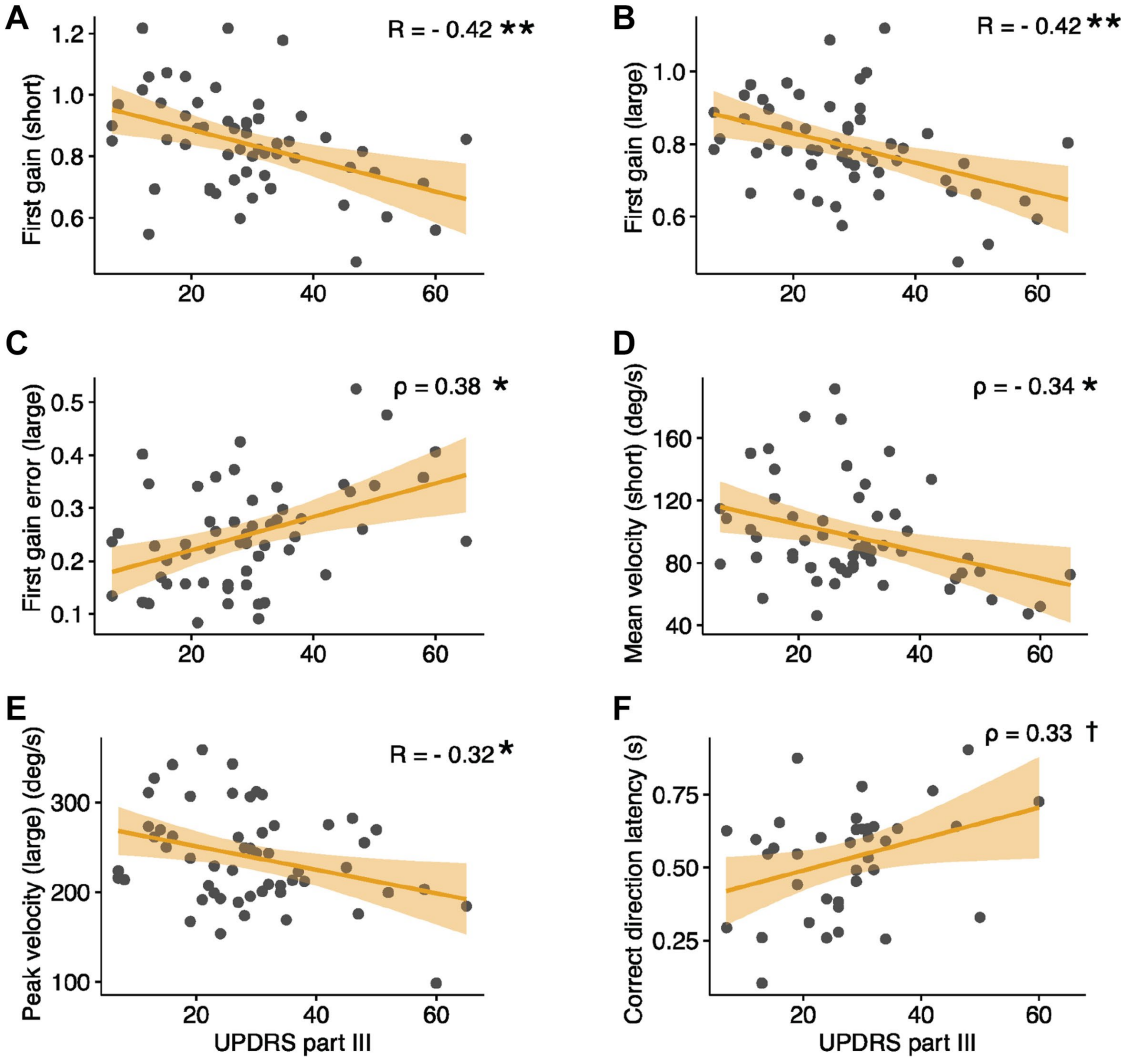
Correlations between select eye-tracking parameters and UPDRS Part III scores. **(A,B)** Pro-Saccades: first gain, **(C)** first gain error, **(D)** mean velocity, **(E)** peak velocity. **(F)** Anti-Saccades: correct direction latency. **(C,D,F)** depict Spearman’s rho values; trend lines are shown for reference only. Large, large amplitude pro-saccades; short, short amplitude pro-saccades. *p < 0.05, **p < 0.01 (corrected for multiple comparisons), ^†^*p* = 0.039 (0.19 corrected).

No fixation parameters were found to correlate with the UPDRS motor score (all *ρ* ≤ 0.187, *p* ≥ 0.542; see Table 2). In contrast, most pro-saccade parameters were found to correlate with it, particularly for large eccentricity targets (see Table 3; Figures 2A–E), six of which survived the value of *p* correction for multiple comparisons (|R| = 0.315–0.419, |*ρ*| = 0.334–377, *p* ≤ 0.028). A single anti-saccade parameter, correct direction latency, was found to correlate with the UPDRS motor score (*ρ* = 0.331, Figure 2F), however, the corrected value of *p* was greater than 0.05.

**Table 2:** Fixation task.

	UPDRS part III correlations		PD/HC comparisons	
Eye-tracking parameter	Correlation coefficient	*p* (corrected)	*F*-statistic	*p* (corrected)
BCEA 68	*ρ* = 0.107	0.425 (0.708)	3.075	0.082 (0.137)
BCEA 95	*ρ* = 0.165	0.217 (0.542)	2.143	0.146 (0.182)
Horizontal gaze SD	*ρ* = 0.187	0.161 (0.542)	3.704	0.056 (0.137)
Vertical gaze SD	*ρ* = 0.02	0.882 (0.978)	0.002	0.964 (0.964)
Saccadic intrusion rate	*ρ* = 0.004	0.978 (0.978)	20.878	1.32 × 10^−5^ (6.6 × 10^−5^)

For each eye-tracking parameter, parameter-UPDRS motor score correlations are shown on the left side of the table (UPDRS part III correlations). Between-group (PD vs. HC) comparisons are shown on the right side of the table (PD/HC comparisons). *ρ*, Spearman’s rho. *F*-statistic and *p*-values for post-hoc analyses are reported. Raw *p*-values are presented, followed by their corrected value in parentheses (Benjamini-Hochberg procedure, *α* = 0.05).

**Table 3:** Prosaccade task.

	UPDRS part III correlations				PD/HC comparisons			
Eye-tracking parameter	Short amplitude		Large amplitude		Short amplitude		Large amplitude	
	Correlation coefficient	*p* (corrected)	Correlation coefficient	*p* (corrected)	*F*-statistic	*p* (corrected)	*F*-statistic	*p* (corrected)
Latency (mean)	*R* = 0.263	0.048 (0.061)	*R* = 0.251	0.059 (0.068)	25.308	1.83 × 10^−6^, (8.5 × 10^−6^)	9.35	0.002 (0.005)
Time to target (mean)	*R* = 0.269	0.042 (0.058)	*R* = 0.341	0.009 (0.02)	6.843	0.01 (0.015)	0.002	0.962 (0.962)
Saccades to target (mean)	*ρ* = 0.153	0.253 (0.253)	*ρ* = 0.196	0.142 (0.152)	28.834	4.21 × 10^−7^ (2.94 × 10^−6^)	45.519	6.54 × 10^−10^ (9.23 × 10^−9^)
First gain (mean)	*R* = −0.416	0.001 (0.007)	*R* = −0.419	0.001 (0.007)	19.278	2.53 × 10^−5^ (8.88 × 10^−5^)	9.829	0.002 (0.005)
First gain (mean error)	*ρ* = 0.371	0.004 (0.014)	*ρ* = 0.377	0.003 (0.014)	2.619	0.108 (0.142)	7.987	0.005 (0.009)
Velocity (mean)	*ρ* = −0.339	0.01 (0.02)	*ρ* = −0.334	0.01 (0.02)	11.376	0.001 (0.002)	0.009	0.924 (0.962)
Peak velocity (mean)	*R* = −0.271	0.04 (0.058)	*R* = −0.315	0.016 (0.028)	2.565	0.112 (0.142)	0.056	0.813 (0.948)

For each eye-tracking parameter, parameter-UPDRS motor score correlations are shown on the left side of the table (UPDRS part III correlations). Between-group (PD vs. HC) comparisons are shown on the right side of the table (PD/HC comparisons). *ρ*, Spearman’s rho. R, Pearson’s R. *F*-statistic and *p*-values for post-hoc analyses are reported. Raw *p*-values are presented, followed by their corrected value in parentheses (Benjamini-Hochberg procedure, *α* = 0.05).

### Group comparisons

**Figure 3:**
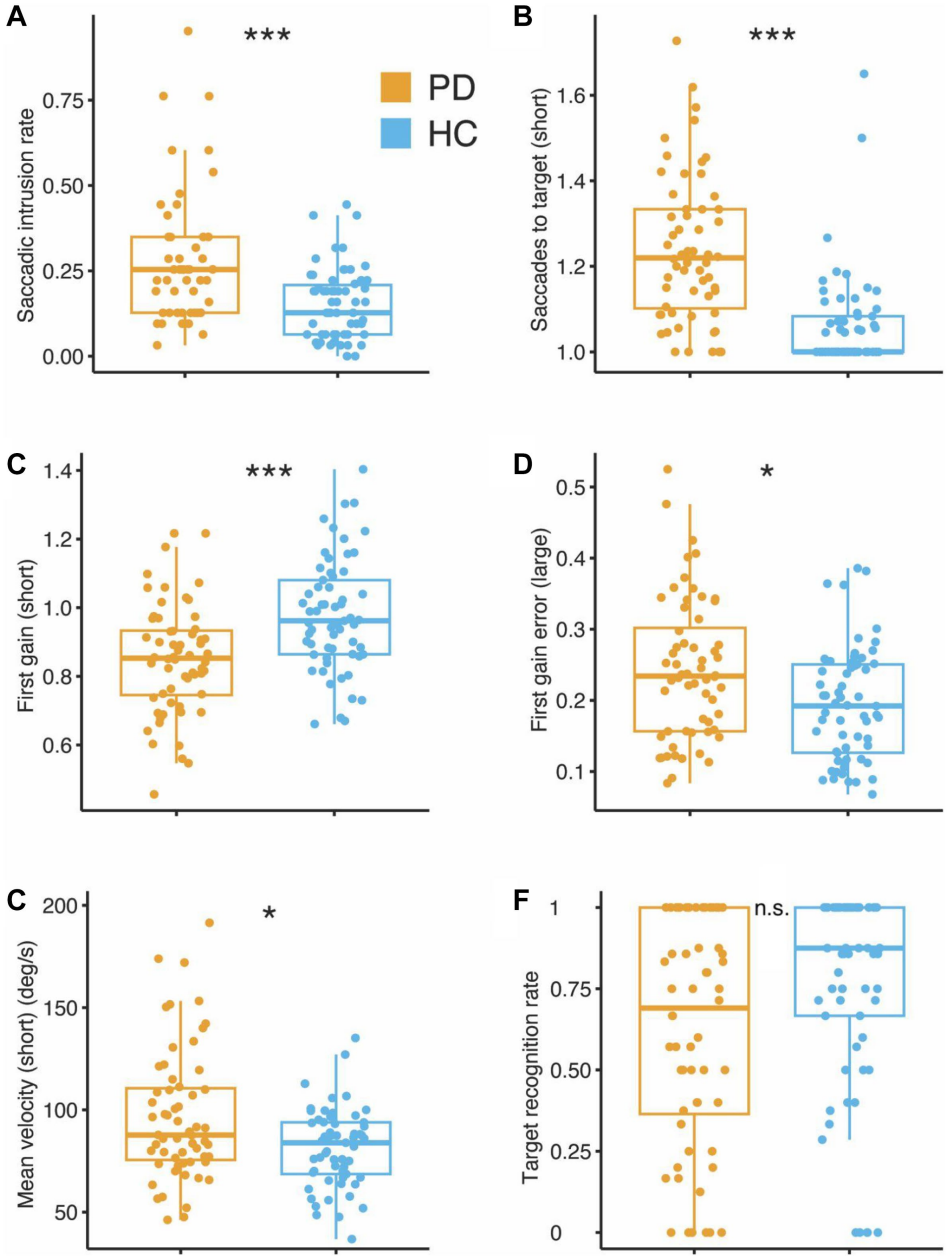
Group differences in eye-tracking parameters between patients with Parkinson’s Disease (PD) and Healthy Controls (HC). **(A)** Fixation: saccadic intrusion rate. **(B)** Pro-Saccades: saccades to target, **(C)** first gain, **(D)** first gain error, **(E)** mean velocity, and **(F)** Anti-Saccades: target recognition rate. Large, large amplitude pro-saccades; short, short amplitude pro-saccades. *p < 0.05, ***p < 0.001 (corrected for multiple comparisons).

For the fixation task, the group effect was significant but the effects of age and sex were not (group: *F*(5) = 5.34, *p* < 0.001; age and sex: both *F*(5) ≤ 1.40, *p* ≥ 0.22, MANCOVA). Only one significant group difference was observed among the fixation parameters, where PD patients displayed a higher saccadic intrusion rate (88.7% average increase in PD, *F*(1) = 20.87, *p* < 0.001; see also Table 2 and Figure 3A).

The effects of age and group were both significant for the pro-saccade task (*F*(5) ≥ 1.90, *p* ≤ 0.034; sex: *F*(5) = 0.62, *p* = 0.84, MANCOVA). Post-hoc analyses yielded several significant group differences (Table 3), particularly those relating to the number of saccades required to reach the target (14.8 and 23% increase in PD, for short and long eccentricities, respectively; Figure 3B), latency (9.7 and 7.2% average decrease in PD, for short and long eccentricities, respectively), saccade precision (13.1 and 9.1% average gain decrease in PD, short and long eccentricities, respectively; Figures 3C,D), and mean saccade velocity (20.6% increase in PD, short eccentricity only; Figure 3E) (all *F*(1) ≥ 6.84, *p* ≤ 0.015).

Finally, we found no significant effects for group, age, or sex for anti-saccade parameters (all *F*(5) ≤ 2.13, *p* ≥ 0.072, MANCOVA; e.g., Figure 3F). Between-group comparisons are reported in Table 4 for transparency, although correcting for multiple comparisons was not deemed necessary in the absence of potential false positives (Type I errors).

**Table 4:** Anti-saccade task.

	UPDRS part III correlations		PD/HC comparisons	
Eye-tracking parameter	Correlation coefficient	*p* (corrected)	*F*-statistic	*p*
Correct saccade (%)	*ρ* = −0.083	0.537 (0.621)	2.448	0.124
Corrected saccade (%)	*ρ* = 0.07	0.621 (0.621)	1.254	0.267
Target recognition rate	*ρ* = −0.189	0.158 (0.365)	2.195	0.145
Correct direction latency (mean)	*ρ* = 0.331	0.039 (0.195)	0.045	0.832
Incorrect direction latency (mean)	*ρ* = 0.173	0.219 (0.365)	0.521	0.47

For each eye-tracking parameter, parameter-UPDRS motor score correlations are shown on the left side of the table (UPDRS part III correlations). Between-group (PD vs. HC) comparisons are shown on the right side of the table (PD/HC comparisons). *ρ*, Spearman’s rho. *F*-statistic and *p*-values for post-hoc analyses are reported.

### Logistic regression classification

**Figure 4:**
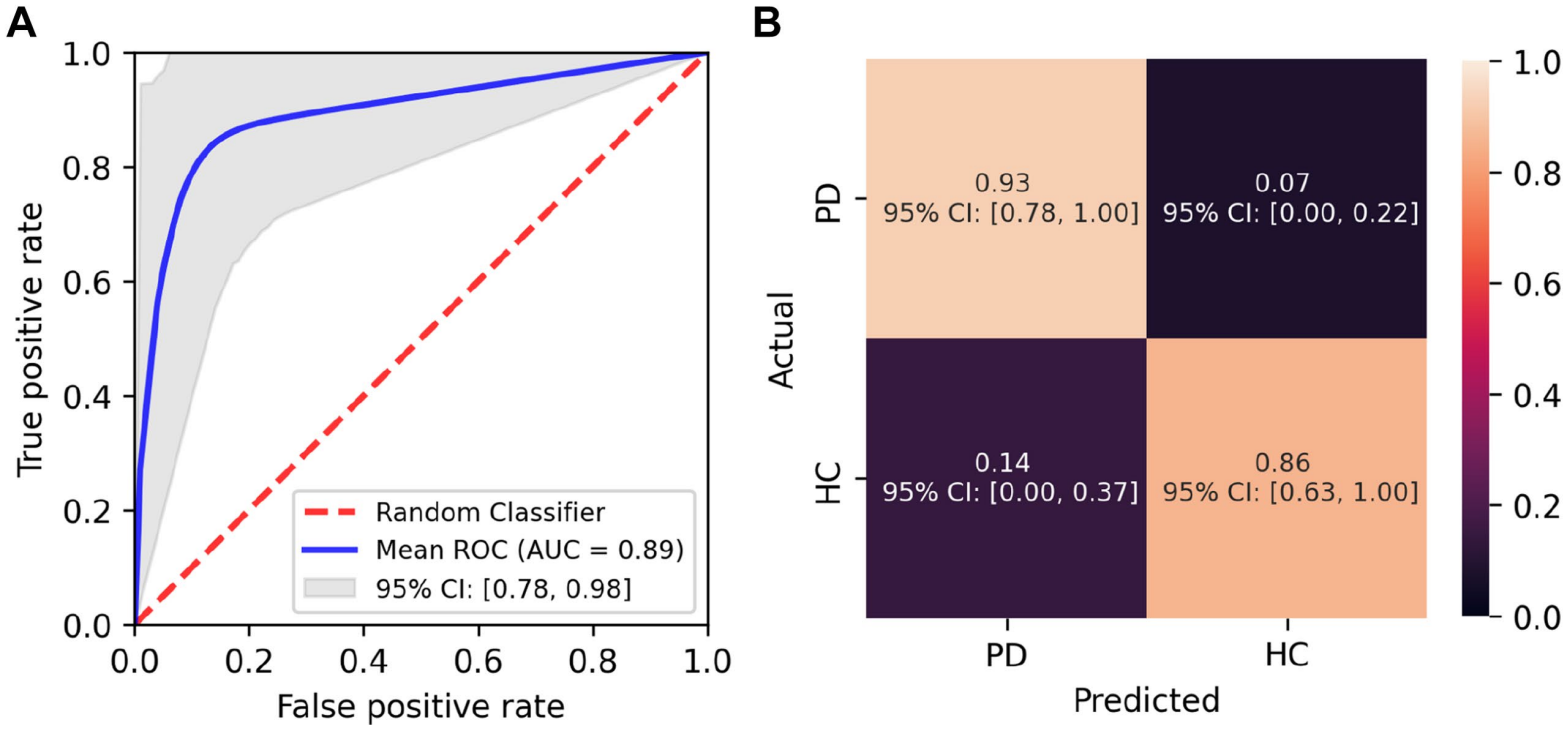
Performance of the logistic regression classifier. **(A)** Mean ROC curve for the logistic regression classifier across random subsamples with 95% confidence interval. **(B)** Confusion matrix for classification of eye tracking parameters as Parkinson’s Disease (PD) and Healthy Controls (HC).

The average receiver operating characteristic (ROC) curve for the logistic regression classifier was computed and had an area under the curve (AUC) of 0.89 (95% CI [0.78,0.98]; Figure 4A). The classifier has a sensitivity of 0.93 (95% CI [0.78, 1.00]) and specificity of 0.86 (95% CI [0.63–1.00]; Figure 4B). Adding age to the logistic regression classifier as an additional parameter did not improve the sensitivity (0.93) or specificity (0.86) of the classifier.

## Discussion

The purpose of the present paper was to demonstrate the potential and usefulness of a novel tablet-based software (currently designed for use with iPad Pros) for the assessment of gaze and eye-movement parameters both in research and clinical practice settings. Not only are our oculomotor findings in line with those previously reported (discussed in greater detail below), but by performing a logistic regression classification, we were able to reliably differentiate individuals with PD from healthy participants based on a subset of the collected eye movement data for which there were statistically significant differences between groups. The AUC (0.89), sensitivity (0.93), and specificity (0.86) metrics obtained are highly comparable to previously published studies on oculomotor anomalies in PD using eye movement parameters extracted with conventional eye-tracking equipment (25, 28, 47, 48). These findings highlight the potential for broader application of eye-movement-based monitoring and early diagnosis technologies.

Individual oculomotor findings are very much in line with those previously reported in the scientific literature on oculomotor anomalies specific to PD. Our finding of an increased rate of saccadic intrusions during fixation confirms previous reports (26, 28, 49, 50). With regards to measures of gaze stability, to our knowledge, only one study reported BCEA and horizontal/vertical gaze SD measures in PD patients and found no significant differences with healthy controls (28).

Pro-saccades have been more extensively studied in PD than measures of fixation instability. Findings regarding saccade latency have been mixed to date, with several studies finding either no differences between PD patients and controls (47, 51–53), shorter latencies in PD (54) and longer latencies in PD (25, 26, 55). It’s unclear why this discrepancy across studies exists, but it may have to do with the type of eye-tracking technology used; the majority of the studies cited above that found either no difference or shorter latencies in PD used infrared eye-trackers from manufacturers such as Eyelink and Tobii, whereas the majority of studies cited above finding increased latencies used devices from other manufacturers such as Micromedical Technologies and Interacoustics, and EyeBrain. To make a more definite statement as to why this would be the case, however, would require a more in-depth investigation that is beyond the scope of the paper.

Similarly, findings regarding peak velocity have been mixed, with a few studies finding faster velocities in PD (24, 56), whereas most other studies found no differences between PD and healthy control (47, 51, 54, 57, 58). In the present study, we find peak and mean velocities to be significantly increased in PD patients (only uncorrected value of *p* for peak velocity) for short eccentricity targets only. Combined with the available literature, this finding suggests that peak velocity might normalize in PD with increasing eccentricity.

In contrast to latency and velocity parameters, the literature is quite rich with strong evidence of PD patients requiring multi-step saccades to reach the target (47, 54, 57, 59), which is in line with the present findings reported here, where the largest group difference amongst all parameters investigated concerned the average number of saccades required to reach the pro-saccade targets. In addition, our findings here indicate that the first saccade towards the target (for both short and large eccentricity targets) was closer to the target in HC.

With regards to anti-saccade parameters, although several studies report a reduced proportion of correct initial direction (or an increase in error rate) in PD patients (25, 52, 60–62), several other studies found no such difference (63–65). Similarly, while several studies have identified slower latencies for correct (52, 61, 63), others found no group differences (60, 62, 66).

Taking a closer look at the reported findings in the literature, it can be observed that many of those studies that identified a difference in the correction direction rate found no differences regarding latency (60, 62, 66), and *vice-versa* (25, 63, 65), indicating either variability in the PD population or that the differences measured could be specific to the anti-saccadic task parameters (e.g., eccentricity of the targets or inter-trial interval). A recent meta-analysis on antisaccade parameters in PD confirmed that, although both antisaccade latency and error rate are significantly increased in PD, these effects are strongly moderated by disease duration and disease severity – as assessed by UPDRS score and H&Y stages (67). This likely explains the absence of significant findings regarding the antisaccade latency and error rate in the present study, as the majority of our PD participants would fall in the mild or moderate category based on their MDS-UPDRS score part III (68).

Few studies to date, to our knowledge, have investigated the relationship between disease severity in PD, such as measured by the MDS-UPDRS motor score and the magnitude of gaze and eye movement parameters. These have primarily observed a relationship between the motor score and pro-saccade latency (26, 48), prosaccade gain (25), anti-saccade latency (24) and anti-saccade direction rate (23). However, (65) found no significant correlation between anti-saccade latency or pro-saccade latency and the UPDRS motor score.

In the present study, we only found a significant UPDRS motor score correlation with pro-saccade gain (large eccentricities) and the number of saccades to reach the target (large eccentricities). We also found a significant correlation between the UPDRS motor score and the pro-saccade time-to-target parameter (large eccentricities), which in many ways represents a composite measure of the latency and the mean velocity of the saccade. With regards to the anti-saccade task parameters, it is unclear why the discrepancies between the cited literature and our study exist. One obvious difference between our PD patient sample is that the error rate was significantly larger in our study [61% vs. 15% in (23)] despite anti-saccade targets being positioned at a similar eccentricity. Finally, a limitation of the present study, however, is the absence of cognitive measures (e.g., MMSE of MoCA) that could have allowed us to further quantify disease severity and investigate associated oculomotor anomalies such as previously done (27–30).

Despite the promise of eye tracking for both research and clinical settings, applications have been limited by the high cost of eye trackers and their inability to scale due to the use of specialized hardware. Being able to make use of the embedded cameras of mobile devices allows us to overcome these cost and scalability barriers by democratizing access to eye-tracking assessment tools. In particular, we believe tablet-based tools have the potential to aid with disease progression monitoring via the assessment of the integrity of the oculomotor system, as demonstrated by the strong relationships found between various eye-movement parameters and clinical status. Such tools could help clinicians monitor changes to disease status, disease progress, or response to treatment remotely without the need for an in-clinic visit until a change in associated eye movement parameters is detected by the software. This approach would be akin to current alternative strategies being developed to remotely monitor motor function & dysfunction with gyroscope/accelerometer-based wearable technologies (69, 70) and speech analysis using machine learning techniques (71, 72).

An advantage of eye-movement-based monitoring technologies, as opposed to wearable technologies, for example, is that they could potentially be more easily scaled to other neurodegenerative disorders. Indeed, as highlighted earlier, several eye-movement anomalies have been tied to AD (11, 14) and MS (8, 18), for instance, and several measured parameters have been shown to highly correlate with their respective cognitive (20–22) or clinical disease scales (31–35).

To conclude, in this study we show that a novel tablet-based eye-tracking technology can reliably identify differences in subtle eye movement abnormalities in PD, and that specific oculomotor parameters were found to significantly correlate with the disease severity stage. Moreover, we were able to reliably differentiate individuals with PD from healthy participants based on a subset of the collected eye movement data. These findings suggest the potential for broader application of eye-movement-based monitoring technologies in neurodegenerative disorders, such as MS and AD, holding promise for their future role in facilitating early diagnosis and monitoring of disease progression. Next steps include validating the technology within a distinct neurodegenerative disorder with known oculomotor impairments and to establish links between oculomotor parameters and clinical measures of cognition. This tablet-based tool has the potential to rapidly scale eye-tracking use and usefulness in both research and clinical settings.

## Data availability statement

The raw data supporting the conclusions of this article will be made available by the authors, without undue reservation.

## Ethics statement

The studies involving human participants were reviewed and approved by The Veritas Independent Review Board and the Montreal University Health Center (MUHC) Research Ethics Board gave ethical approval of this work. The patients/participants provided their written informed consent to participate in this study.

## Author contributions

ÉV-S and SD: conceptualization and supervision. PV, JC-F, and NK: formal analysis. ÉV-S: funding acquisition. JC-F and SD: investigation. ÉV-S, PV, and DG: methodology. JC-F and NK: visualization. PV: writing – original draft preparation. ÉV-S, PV, DG, JC-F, and SD: writing – review and editing. All authors contributed to the article and approved the submitted version.

## Funding

This project was supported by Natural Science and Engineering Research Council of Canada, grant RGPIN-2019-04761.

## Conflict of interest

ÉV-S is a co-founder of Innodem Neurosciences, which developed the Eye-Tracking Neurological Assessment (ETNA™) technology used in this study. PV has ownership options in Innodem Neurosciences. JC-F is a part-time employee of Innodem Neurosciences and NK is a research intern at Innodem Neurosciences. SD has previously served as an advisor to Innodem Neurosciences.

The remaining author declares that the research was conducted in the absence of any commercial or financial relationships that could be construed as a potential conflict of interest.

## Publisher’s note

All claims expressed in this article are solely those of the authors and do not necessarily represent those of their affiliated organizations, or those of the publisher, the editors and the reviewers. Any product that may be evaluated in this article, or claim that may be made by its manufacturer, is not guaranteed or endorsed by the publisher.
